# Nodular glomerulosclerosis in patients’ without history of diabetes mellitus: a case report

**DOI:** 10.4076/1757-1626-2-6792

**Published:** 2009-09-16

**Authors:** Imed Helal, Rym Goucha, Cyrine Karoui, Ezzedine Abderrahim, Fethi Ben Hamida, Fethi Elyounsi, Hedi Ben Maiz, Taieb Ben Abdallah, Adel Kheder

**Affiliations:** Department of Internal Medicine A and Laboratory of Kidney Pathology 02, Charles Nicolle Hospital Boulevard 9 Avril. 1006 TunisTunisia

## Abstract

**Introduction:**

Diabetic nephropathy can occur during the course of both type1 and type 2 diabetes mellitus. The characteristic lesions are diffuse or nodular (Kimmelsteil-Wilson) diabetic glomerulosclerosis. The reported cases represent unusual presentations of diabetes mellitus.

**Case presentation:**

We report the case of a 49-year-old man without prior history of diabetes mellitus who presented with rapidly progressive renal failure and whose renal biopsy revealed nodular (Kimmelsteil-Wilson) glomerulosclerosis lesions characteristic of diabetes.

**Conclusion:**

Renal manifestations of diabetes mellitus may antedate other more common presenting symptoms of this disease and we critically review the literature on this subject.

## Introduction

The incidence and prevalence of diabetes mellitus (DM) are increasing. Tunisia, like most countries of the world, is experiencing an alarming rise in the number of people with diabetes. The prevalence of types 2 diabetes in adult over 30 year of age rose from 4.2% in 1976 to 10% in 1995 [1]. The situation is similar in other countries. Nowadays, altogether 120 million people are diabetic in the world and the number will triple in 30 years. The diabetes-related medical cost is increasing [2]. DM has become an enormous social problem. Accordingly, the prevalence of diabetic nephropathy (DN) is also increasing. It has become the leading cause of end-stage renal diseases (ESRD) in developed countries. By USRDS reports [3], the number of incident patients with diabetes as their primary cause of renal failure will continue to increase though the growth rate has slowed a little bit. In Tunisia, it is turning out to be a major cause of ESRD, diabetic nephropathy rising at the rate of 16.1% yearly [3,4].

## Case presentation

We report the case of a 49-year-old Arabic Tunisian man without prior history of diabetes mellitus who presented with rapidly progressive renal failure. Her medical history was otherwise non-contributory. He denied the usage of any drugs.

On physical examination, he was afebrile, with a blood pressure of 120/70 mm Hg and a pulse of 100 beats/min without orthostatic changes. The chest test was normal and he had no murmurs or rubs. He had 4+ oedema of his lower extremities. His skin, scalp, oral mucosa, and genitals were free of rash or other lesions. The remainder of his physical examination was unremarkable.

His blood urea nitrogen level was 33.6 mmol/l and creatinine level was 613 µmol/l, where as electrolyte levels were normal. Glucose level was 5.1 mmol/l and uric acid was 755 µmol/l. Serum protein electrophoresis confirmed the presence of hypoalbuminemia (22.8 g/l), the total protein was 56 g/l and demonstrated polyclonal elevation of the gamma-globulins (13 g/l). The cholesterol was 4.1 µmol/l and triglycerides was 1.1 mmol/l. Liver function tests were normal. A 24-hour urine collection showed 4.74 g of protein. Urine microscopy showed the presence of red blood cells (70000/ml). Haemoglobin was 7.1 g/dl and hematocrit was 23%, white blood cell count was 8000/µl, and platelets were 325000/µl. coagulation study results were normal.

Serological evaluation included negative test results for hepatitis B and C, serum antinuclear antibodies, antineutrophil cytoplasmic antibody (ANCA) and anti-glomerular basement membrane. C3, C4 and CH50 were all in normal ranges. There was no evidence of dysproteinemia on serum or urine electrophoresis. Ultrasonography showed normal-sized kidneys with normal echogenicity.

Regarding the history, the renal function worsened rapidly from 321 to 613 µmol/l in few days. The urine was 3+ for protein and 2+ for hematuria. Our patient did not have a prior diagnosis of DM or other clinical or biological manifestations of other systemic disease which could be responsible for the nephropathy. These findings led to the diagnosis of rapidly progressive glomerulonephritis and realization of renal biopsy.

The patient was diagnosed as having rapidly progressive glomerulonephritis and he underwent an ultrasonography-guided renal biopsy. Light microscopic examination demonstrated thickening of the glomerular capillary basement membranes with expansion of the mesangial area and hyalinosis of same afferent and efferent arterioles. Immunofluorescence did not show anything significant. Electronic microscopy was not performed. The renal biopsy specimen revealed nodular (Kimmelsteil-Wilson) glomerulosclerosis lesions characteristic of diabetes. Therefore, we complete investigation by fundoscopic ophthalmoscopy witch revealed grade 2 diabetic retinopathy.

After further evaluation, we don’t found classical progression factors of diabetic nephropathy. The patient has end-stage renal disease, so he actually was regular dialyzed in our department.

## Discussion

Diabetic nephropathy can occur during the course of both type 1 and type 2 diabetes mellitus. The characteristic lesions are diffuse or nodular (Kimmelsteil-Wilson) diabetic glomerulosclerosis. It’s the most frequent cause of renal disease in patients with type II diabetes mellitus (DM), sometimes accompanied by vascular lesions.

Our patient did not have a prior diagnosis of DM or other clinical or biological manifestations of other systemic disease which could be responsible for the nephropathy. However, her renal biopsy revealed nodular glomerulosclerosis which might indicate an association with DM. In addition, the diabetic retinopathy found in fundoscopic ophthalmoscopy confirmed the diagnosis of diabetic nephropathy. Until now, only few cases have been reported with this presentation.

Diabetes duration is an indicator of DN in type 2 DM. Patients with persistent proteinuria and a relatively short period of diabetes should be examined carefully to identify non-diabetic renal diseases (NDRD). In DN, it often takes quite a long period of time to go from micro-albuminuria to macro-albuminuria, and even renal failure. DN is one of the chronic complications of diabetes. Clinical abnormalities are often detected 5-10 years after onset or diagnosis of DM. The patient with relatively shorter diabetes duration is probably thought to be NDRD.

Diabetic retinopathy (DR) is one of the microvascular complications of DM, which might have the same pathogenetic pathways as DN. Retinopathy, when it coexists with nephropathy (usually called renal-retinal syndrome), is thought to be a window of renal complication. Diabetic retinopathy may serve as an indicator of DN. The relationship between retinopathy and nephropathy in type 1 DM has been demonstrated in some studies [4-6]. In type 2 DM, it was confirmed by Fioretto’s cohort [7] that almost all microalbuminuric patients had DR and all patients with proliferative DR had typical DN. Results of Parving *et al*. [7,8] showed that all the proteinuric DM patients with DR had DN. On the other hand, Parving believed that lack of DR was a poor predictor of NDRD since the chance for DN or NDRD was fifty-fifty. The reported cases represent diabetic nephropathy as the mode of presentation of diabetes mellitus. Renal manifestations of diabetes mellitus may antedate other more common presenting symptoms of this disease [8,9]. The clinical course of patients with diabetic glomerulosclerosis seems to resemble that of rapidly progressive glomerulonephritis, so renal biopsy must be performed.

Though many indicators have been found to be important distinguishing DN from NDRD in the literature, it is still unknown how to identify DN effectively, safely and scientifically. Kidney biopsy is the most effective method to identify DN in type 2 DM, but it can not be performed on all the patients due to factors such as anticoagulation, active bleeding, unilateral nephrectomy or reluctant to biopsy. Basically, people used to believe that a biopsy had to be taken to clinically diagnose DN. The diagnosis criteria were developed as follows: persistent albuminuria, presence of diabetic retinopathy and absence of any clinical or laboratory evidence of other kidney or renal tract disease [7,8]. Also, Glassock [9,10], had presented biopsy criteria previously, but Serra [10,11] thought these biopsy criteria were not useful in identifying patients with other renal diseases. Based on this, some researchers investigated the frequencies of NDRD in diabetic patients, and the results varied. The inclusion criteria may play a role and derive conflicting conclusions.

## Conclusion

The reported cases represent diabetic nephropathy as the mode of presentation of diabetes mellitus. Renal manifestations of diabetes mellitus may antedate other more common presenting symptoms of this disease. We critically review the literature on this subject, pointing out the pitfalls in diagnosis and establishing strict criteria for the diagnosis of diabetic nephropathy in patients without overt clinical diabetes.

**Figure 1.**
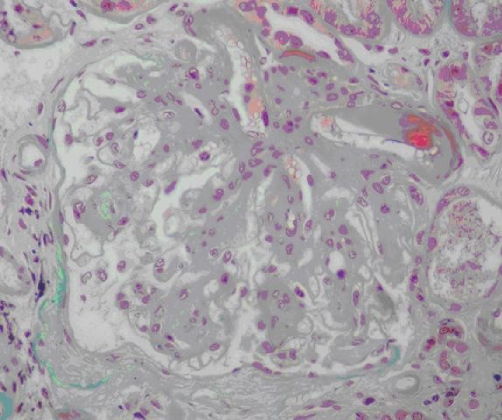
Nodular (Kimmelsteil-Wilson) glomerulosclerosis lesions characteristic of diabetes (Masson trichrome coloration).

**Figure 2.**
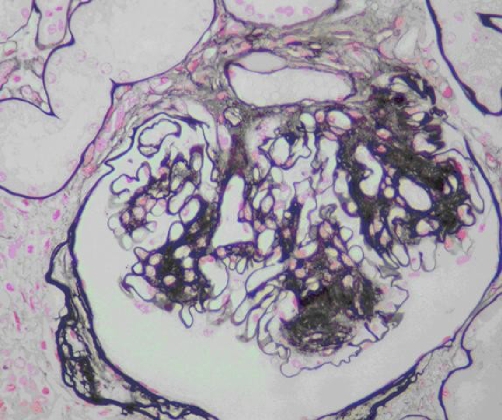
Glomerulosclerosis lesions characteristic of diabetes (Argentic coloration).

## Abbreviations

ANCA: antineutrophil cytoplasmic antibody
DM: diabetes mellitus
DN: diabetic nephropathy
DR: diabetic retinopathy
ESRD: end-stage renal diseases
